# Detection and phylogenetic analyses of spike genes in porcine epidemic diarrhea virus strains circulating in China in 2016–2017

**DOI:** 10.1186/s12985-017-0860-z

**Published:** 2017-10-10

**Authors:** Qiaoling Zhang, Xinsheng Liu, Yuzhen Fang, Peng Zhou, Yonglu Wang, Yongguang Zhang

**Affiliations:** 1 State Key Laboratory of Veterinary Etiological Biology, OIE/National Foot and Mouth Disease Reference Laboratory, Key Laboratory of Animal Virology of Ministry of Agriculture, Lanzhou Veterinary Research Institute, Chinese Academy of Agricultural Sciences, Lanzhou, 730046 China; 2 Jiangsu Co-innovation Center for Prevention and Control of Important Animal Infectious Diseases and Zoonoses, Yangzhou, 225009 China

**Keywords:** Pedv, Spike gene, Phylogenetic analysis, China

## Abstract

**Background:**

Large-scale outbreaks of porcine epidemic diarrhea (PED) have re-emerged in China in recent years. However, little is known about the genetic diversity and molecular epidemiology of field strains of PED virus (PEDV) in China in 2016–2017. To address this issue, in this study, 116 diarrhea samples were collected from pig farms in 6 Chinese provinces in 2016–2017 and were detected using PCR for main porcine enteric pathogens, including PEDV, porcine deltacoronavirus (PDCoV), porcine transmissible gastroenteritis virus (TGEV) and porcine kobuvirus (PKV). In addition, the complete S genes from 11 representative PEDV strains were sequenced and analyzed.

**Results:**

PCR detection showed that 52.6% (61/116) of these samples were positive for PEDV. Furthermore, sequencing results for the spike (S) genes from 11 of the epidemic PEDV strains showed 93–94% nucleotide identity and 92–93% amino acid identity with the classical CV777 strain. Compared with the CV777 vaccine strain, these strains had an insertion (A^133^), a deletion (G^155^), and a continuous 4-amino-acid insertion (^56^NNTN^59^) in the S1 region. Phylogenetic analysis based on the S gene indicated that the 11 assessed PEDV strains were genetically diverse and clustered into the G2 group. These results demonstrate that the epidemic strains of PEDV in China in 2016–2017 are mainly virulent strains that belong to the G2 group and genetically differ from the vaccine strain. Importantly, this is the first report that the samples collected in Hainan Province were positive for PEDV (59.2%, 25/42).

**Conclusions:**

To our knowledge, this article presents the first report of a virulent PEDV strain isolated from Hainan Island, China. The results of this study will contribute to the understanding of the epidemiology and genetic characteristics of PEDV in China.

## Background

Porcine epidemic diarrhea (PED), which is caused by PED virus (PEDV), is an acute and highly contagious enteric disease in swine characterized by watery diarrhea and vomiting. From 1984 to early 2010, PEDV infections occurred in the pig population in China, but there were no large-scale outbreaks [1, 2]. However, a large-scale outbreak of PED occurred at the end of 2010 and led to the most severe PED epidemic in history [3].

PEDV is an enveloped, single-stranded, positive-sense RNA virus that belongs to the order *Nidovirales*, the family *Coronaviridae* and the genus *Alphacoronavirus* [4]. This virus’s spike (S) protein, which is a glycosylated protein located on the envelope of the virus, consists of S1, a receptor-binding subunit, and S2, a membrane fusion subunit. The S1 domain is important for recognizing and binding to cell receptors [5, 6]. In addition, the S1 domain contains several neutralizing epitopes that stimulate the induction of neutralizing antibodies in the natural host [7, 8]; therefore, the S protein has been the primary target for the development of vaccines against PEDV and for determining genetic relatedness among PEDV isolates.

In this study, a total of 110 diarrheal fecal samples and 6 intestinal contents samples were collected from swine farms in six Chinese provinces in 2016–2017. Samples were evaluated using RT-PCR and TaqMan real-time RT-qPCR (set up in our laboratory). To determine the frequency of co-infection, we also assessed samples for other enteric pathogens, such as PDCoV, TGEV and PKV, that are closely linked to porcine diarrhea. The complete S genes from 11 representative PEDV strains were amplified and sequenced, and the GenBank accession numbers for CH/HNXJ/2017, CH/HN7/2016, CH/GSTS/2016, CH/HNXY/2017, CH/HNCD/2017, CH/HNHB/2017, CH/XJKT/2016, CH/HNLB/2017, CH/HNPJ/2017, and CH/SXXY/2017 are MF152596, MF152597, MF152598, MF152599, MF152600, MF152601, MF152602, MF152603, MF152604, and MF152605, respectively. Seventy-four reference strains were obtained from GenBank for phylogenetic analysis. A phylogenetic tree was constructed using the neighbor-joining method and MEGA version 6 [9], with bootstrap values calculated for each node from 1000 replicates. Analysis of amino acid sequences was conducted using DNAStar software (Version 3.1; DNASTAR, Madison, WI, USA).

## Results and discussion

Detection results showed that 52.6% (61/116) of the samples were positive for PEDV. The positive rate for porcine kobuvirus (PKV) in the samples was 19.8% (23/116), and the rate of co-infection with PEDV and PKV was 11.2% (13/116). However, porcine delta coronavirus (PDCoV) and porcine transmissible gastroenteritis virus (TGEV) were not detected in the samples. Rates of positivity for PEDV in Gansu, Xinjiang, Henan, Hainan, Hunan and Shanxi provinces were 16.7% (1/6), 75.0% (3/4), 37.5% (12/32), 59.5% (25/42), 59.2% (16/27), and 80.0% (4/5), respectively. PEDV-positive samples were detected for the first time in Hainan Province in China.

Sequence analysis results showed that all field strains shared 97–100% nucleotide and amino acid identity with each other as well as 93–94% nucleotide identity and 92–93% amino acid identity with the CV777 strain. Compared with the CV777 vaccine strain, the strains in this study had an insertion (A^133^) and a deletion (G^155^); in addition, all of the isolated strains except CH/HNZZ47/2016 had a continuous 4-amino-acid insertion (^56^NNTN^59^), whereas the CH/HNZZ47/2016 strain had a continuous 2-amino-acid insertion (^58^TN^59^). Mutant sites in these strains were primarily located in the S1 domain of the S protein. Moreover, the three epitope regions in CV777 (aa 498–637, aa 747–754 and aa 763–770) were examined to assess mutations in the field strains. We found that the amino acid sequence at aa 747–754 was conserved between the latest field strains and CV777; however, certain mutated regions were observed in the sequences at positions 498–637 and 747–754 (Table 1).

**Table 1:** Analysis of amino acid mutations in epitopes domains of field strains and the CV777 vaccine strain (aa 498–637, aa 747–754, aa 763–770)

Strains	499	520	523	524	545	565	566	569	597	604	611	629	637	766	767	769
CV777	I	A	G	H	D	S	K	D	G	F	S	T	Q	P	L	D
CH/HN7/2016		S							S				E		S	S
CH/HNXJ/2017	T	V	D						S				E	L	S	S
CH/HNCD/2017	T	S							S				E		P	S
CH/HNHB/2017		S					N		S	L			E		S	S
CH/XJKT/2016	T	V							S				E	L	S	S
CH/HNLB/2017	T		D	R			V	V	S				E		S	S
CH/HNPJ/2017	T			L					S				E		S	S
CH/GSTS/2016		S			S				S		G	I	E		S	S
CH/SXXY/2017		S							S				E		S	S
CH/HNXY/2017	T	S		R			N		S				E		S	S
CH/HNZZ47/2016		S				F			S				V		S	S

**Fig. 1:**
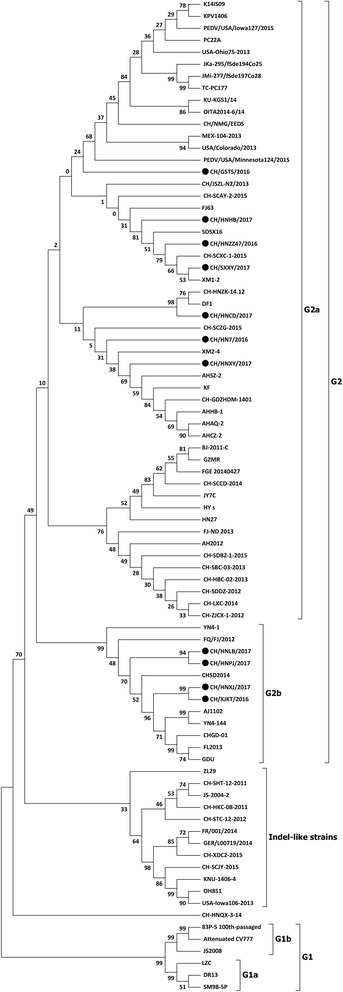
Phylogenetic analysis using the S genes of 74 PEDV reference strains and the 11 PEDV isolates in our study. The tree was constructed using the neighbor-joining method, with bootstrap values calculated from 1000 replicates using MEGA 6 software. “●” indicates the isolates in our study

In the phylogenetic tree based on the S gene, the S protein-encoding genes indicated that all 11 PEDV epidemic strains were of subtype G2 (Fig. 1). More specifically, CH/HNXY/2017, CH/HNCD/2017, CH/HNHB/2017, CH/SXXY/2017, CH/HN7/2016, CH/GSTS/2016 and CH/HNZZ47/2016 were categorized as subtype G2a, whereas CH/HNLB/2017, CH/HNXJ/2017, CH/HNPJ/2017, and CH/XJKT/2016 were categorized as subtype G2b (Fig. 1). In contrast, early PEDV isolates in China, such as CH/S, LZC, and the CV777 vaccine strain, are classical G1 strains.

**Fig. 2:**
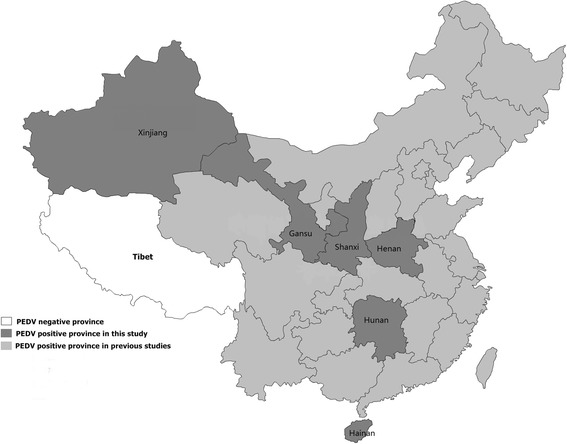
PEDV distribution map in China

In this study, the rate of positivity for PEDV in China in 2016–2017 exceeded 50%, indicating that PEDV has remained prevalent in China; this rate was significantly higher than those determined for earlier PEDV outbreaks in China. Although no previous reports indicated that PEDV existed in Hainan Province, in this study, PEDV was detected in samples collected from that location (with a positive rate of 59.2% (25/42)). This result indicates that PEDV has spread to Hainan Province, with positivity for PEDV detected in a high proportion of clinical samples from that province. Therefore, PEDV infection is trending toward becoming a national epidemic in China. PEDV positive provinces of China was shown in Fig. 2.

Currently, many enteric viruses are known to induce porcine diarrhea, including PEDV, TGEV, PKV, PDCoV, and porcine rotavirus (PRoV), which are closely linked to porcine diarrheal disease. An epidemiological study conducted in 2012–2014 showed that co-infection with PKV and PEDV was detected in 15.0% (47/314) of samples and that co-infection with PKV and one or more of TGEV, PDCoV, and PRoV was common [10]. In this study, the rate of co-infection with PEDV and PKV was 11.2% (13/116) in diarrhea samples. However, co-infection with PEDV and TGEV or PDCoV was not detected. Additionally, the rate of PEDV positivity was 52.6% (61/116) for samples from 2016 to 2017. The infection rate in the assessed samples was higher for PEDV than for other diarrhea viruses; this result was consistent with the findings of certain prior epidemiological surveys conducted in China [10–12]. Thus, the aforementioned results indicate that PEDV remained the main pathogen causing swine diarrhea in China.

The S gene is a significant structural gene of PEDV and is thought to encode the most antigenic protein for inducing neutralizing antibodies against PEDV [13, 14]. Therefore, the S gene is used to analyze the molecular epidemiology and genetic relatedness of PEDV. In this study, the 11 field strains were classified into the G2 group according to a phylogenetic tree. This result indicated that virulent strains were prevalent in China and were responsible for PEDV outbreaks in 2016–2017. In addition, to an extent, the examined strains differed from early PEDV isolates (LZC, CH/S and CV777). This difference explains why PED outbreaks continued to occur in cases involving the use of CV777-based vaccines. Based on the amino acid sequence of the S gene, the mutant sites in these strains were primarily located in the S1 domain of the S protein. The CV777 vaccine strain has three major neutralizing epitopes: aa 498–637 (the COE gene), aa 747–754 (YSNIGVCK) and aa 763–770 (LQDGQVKI) [2, 14]. We found that strains in this study had sequence mutations at positions 498–637 and 747–754; however, the amino acid sequence at aa 747–754 was conserved between the field strain most recently isolated in our study and CV777. This result is consistent with the observation that CV777-based vaccines provided partial cross-protection against PEDV epidemic strains in China. In summary, current field strains of PEDV found in China in 2016–2017 are primarily virulent strains, and genetic variations in these strains may have led to antigenic changes and the failure of immunization.
